# Supplementary material for: Predictors of diarrhea among children under 24 months in Nepal: A multilevel analysis of multiple indicator cluster survey 2019
Source: PLoS One. 2023 Jul 6;18(7):e0287974. doi: 10.1371/journal.pone.0287974 (PMC10325074; doi:10.1371/journal.pone.0287974)
Supplement: Attachment — Submitted filename: Response to Reviewers.docx [file pone.0287974.s001.docx]

**Reviewer #1: Major comments**

Reviewer: The rationale for the study is not strong enough. Especially, what is the gap in previous literature about the factors associated with childhood diarrhea among children aged 0-24 months? Or in another words, is this topic previously studied or not? If yes, what is the gap you are trying to fulfill? I recommend you to provide literature review related to your study and provide gaps in the literature, and show the contribution made by your study. You can address this issue in your fourth paragraph of introduction section.

Authors: Thank you very much for the suggestion. We have added the current literature and gaps in the introduction section.

Reviewer: The authors said that they categorized the factors into three categories as presented in Figure 1. Is the conceptual framework based on previous study or any standard framework? If yes, please provide the reference.

Authors: Thank you very much for the question. The conceptual framework is not based on solely the previous study or framework. It is based on an extensive literature review. After the extensive literature review, we categorized the significant variable available in the dataset into three categories as presented in the Figure 1. We have added few lines in the conceptual framework subsection to make it clearer.

Reviewer: In terms of vaccination status, I recommend to be specific about vaccines that prevent diarrhea. For example, Rota virus vaccine, Rota virus is the most common cause of diarrhea in low- and middle-income countries. I recommend to analyze your data using such vaccines, whether you see any change in p-value? If you observe any difference, you can include it in your model, otherwise, you can describe it in the method section stating that we also analyzed our data using the vaccination status of vaccines that prevent diarrhea.

Authors: Thank you very much for the suggestion. The data was taken from the Multiple Indicator Cluster Survey 2019, where there is a variable named “Rotavirus vaccine ever given”. Under this variable, it is mentioned that 27 under 24-months children were given Rotavirus vaccination, 358 under 24-month children were not given Rotavirus vaccination, 142 reported “don’t know”, 1 didn’t respond and 2039 observations were missing. Therefore, in order to avoid possible unreliable parameter estimation and larger confidence interval due to inadequate sample size for the Rotavirus vaccine ever given variable, the authors did not include this variable in the study.

Reviewer: Is there any reason to include wasting only? Why you did not include stunting and underweight? Please describe about it while describing your study variables.

Authors: Thank you very much for the question. After the extensive literature review, we found that wasting among these three indicators was the most appropriate factor responsible for childhood diarrhea. Wasting was the leading risk factor responsible for 80.4% (95% UI 68.2–85.0) of diarrhea deaths in children younger than 5 years according to recent studies. Therefore, we included only wasting as the indicator of malnutrition to make the model parsimonious as mentioned in the analysis subsection.

Reference: Troeger, C.; Blacker, B.F.; Khalil, I.A.; Rao, P.C.; Cao, S.; Zimsen, S.R.; Albertson, S.B.; Stanaway, J.D.; Deshpande, A.; Abebe, Z.; et al. Estimates of the Global, Regional, and National Morbidity, Mortality, and Aetiologies of Diarrhoea in 195 Countries: A Systematic Analysis for the Global Burden of Disease Study 2016. *Lancet Infect. Dis.* **2018**, *18*, 1211–1228, doi:10.1016/S1473-3099(18)30362-1/ATTACHMENT/35A61B31-B1D1-4B4D-9020-

Reviewer: Please provide reference while categorizing caste ethnic group into two groups: relatively advantaged and relatively disadvantaged.

Authors: Thank you very much for the suggestion. It was categorized based on the report: Trends and determinants of neonatal mortality in Nepal: Further analysis of the Nepal Demographic and Health Surveys, 2001-2011. The categorization criteria have been reported on page 5 of before mentioned document. We have added the reference in the manuscript.

Reference: Paudel D, Thapa A, Shedain P, Paudel B. Trends and determinants of neonatal mortality in Nepal: Further analysis of the Nepal Demographic and Health Surveys, 2001-2011. 2013.

Reviewer: You said that those variables that have p-value less than 0.2 were included in multivariate analysis. Could you provide any reference that recommend this concept? or is this arbitrary cut-off?

The level of significance was set at 0.05 for both bivariate and multivariable analysis. However, we selected the variables which had p-value less than 0.2 in bivariate analysis to be included in the multivariable analysis. It was done so that the important variables which were not significant at traditional level of significance (0.05) could also be included in the model. The fit statistics of models were found to be more appropriate when including variables with p-value less than 0.2 in the model in comparison to model with variables having p-value less than 0.05. Moreover, a study conducted by Bursac et al., has discussed that in order to improve the chances of retaining meaningful confounders, the confounding level can be set above 5%.

Reference: Bursac, Z., Gauss, C.H., Williams, D.K., Hosmer, D.W., 2008. Purposeful selection of variables in logistic regression. Source Code Biol. Med. 2008 31 3, 1–8. https://doi.org/10.1186/1751-0473-3-17

Reviewer: I think your final model is model 2, but it is not clear in data analysis section, please make it clear in words.

Authors: Thank you very much for your comment. Yes, model 2 is the final model. We have split the sentence and added some words to make it clear in the data analysis section.

Reviewer: In line number 238: You provided association of GDP and diarrhea to explain the possible linkage of Province and incidence of diarrhea. Please support your argument with reference that shows the association between GDP and incidence of diarrhea.

Authors: Thank you very much for your suggestion. We have added the statement that shows the association between GDP and the incidence of diarrhea in the discussion section to support our argument.

Reviewer: What are the policy implications of your study? You only talked about it in conclusion section, I think you need a separate subsection for policy implications of your findings. What are the 2-3 key points that policymakers can take from your study?

Authors: Thank you very much for the suggestion. As per your suggestion, we have added a different subsection for the policy implication of our study above the limitation section.

**Reviewer #1: Minor comments**

Reviewer: In table 4, please use reference instead of "1" as a reference category.

Authors: As per your suggestion, we have changed 1 to reference in table 4.

Reviewer: Title of table 2: Characteristics of children under 24 months in seven provinces of Nepal. It does make sense to use "seven provinces of Nepal". You can simple use sample characteristics or sample characteristics of children under 24 months.

Authors: We have changed the title of tables as per your suggestion.

**Reviewer #2:** Manuscript is well written by utilizing the data from the open access data of MICS conducted in Nepal. I suggest authors to more minutely discuss the "participation and sample size " section as it looks inadequate and incomplete. Please specify "what was the basis for identifying numbers of urban and rural strata? you have mentioned "Within each stratum, primary 81 sampling units (PSUs) were selected systematically with probability proportional to size." how did you decide to take 81 PSUs? how did you calculated the sample size? please elaborate.

Author: Thank you very much for the question. As per your suggestion, we have added few lines under “Participants and Sample Size” to make it more clearer, Since, our study adopted the dataset from Multiple Indicator Cluster Survey 2019, which was conducted by the Central Bureau of Statistics, the sampling and sample size calculation was performed by Central Bureau of Statistics, National Planning Commission, Government of Nepal. A multi-stage, stratified cluster sampling approach was used for the selection of the survey sample. The urban and rural areas within each province were made main sampling strata. Rural municipalities represent the rural area whereas the municipalities, sub-metropolitan, and metropolitan cities represent the urban area. Within each stratum, primary sampling units (PSUs) were selected systematically with probability proportional to size. The PSUs selected at the first stage were the enumeration areas (EAs) defined for the census enumeration. A listing of households was conducted in each sample EA, and a sample of households was selected at the second stage.

Since the overall sample size for the Nepal MICS partly depends on the geographic domains of analysis, the distribution of EAs and households in Nepal from the 2011 Census sampling frame was first examined by province, urban and rural strata. As In the survey total of 14,805 women of age 15-49 years were interviewed, which included 6658 mothers/caretakers of children under 5 years. After receiving the dataset, observations of children above 23 months were excluded and 2566 observations out of 6658 mothers/caretakers were retained. Furthermore, 218 observations for different variables with missing data were removed and 2348 observations nested into 512 PSUs were included in the final analysis.

Reference: Central Bureau of Statistics (CBS), 2020. Nepal Multiple Indicator Cluster Survey 2019, Survey Findings Report. Kathmandu, Nepal: Central Bureau of Statistics and UNICEF Nepal.

͕
