# Supplementary material for: Predictors of diarrhea among children under 24 months in Nepal: A multilevel analysis of multiple indicator cluster survey 2019
Source: PLoS One. 2023 Jul 6;18(7):e0287974. doi: 10.1371/journal.pone.0287974 (PMC10325074; doi:10.1371/journal.pone.0287974)
Supplement: Attachment — Submitted filename: Response to Reviewers.docx [file pone.0287974.s002.docx]

Dear reviewer, Thank you for your valuable comments. We have addressed all the comments and incorporated your suggestion in the revised version of the manuscript.

Comments:
1. The sentence mentioned below is very strong as if there are no studies that explored diarrheal risk in under two children. Please revise your sentence because there are some studies that have explored heterogeneity in diarrheal risk across age groups while studying diarrhea in under five children.
"Although previous studies reported that the diarrheal risk is higher in under two years children, no studies have been conducted in this specific population"
It could be: To the extent of our knowledge, we did not find national-level studies that explicitly explored diarrheal risk in under two years children in Nepal.

Authors: Thank you very much for your suggestion. We have changed the sentence according to your suggestion in line number 74-76.

2. As suggested in first review, I think you have not provided the reference for determining the p-value of <0.2?
"First, bivariate analysis was conducted where the Rao-Scott chi131 square test was applied to determine the p-value between diarrhea and independent variables, and variables significant at p<0.2 in bivariate analysis were considered candidate variables for the multilevel analysis"

Authors: Thank you very much for your suggestion. We have added the reference as per your suggestion. The words “variables significant at p<0.2 in bivariate analysis were considered candidate variables for the multilevel analysis” have been changed to “variables having p-value less than 0.2 in bivariate analysis were considered candidate variables for the multilevel analysis” in lines number 132 and 133 in the revised version of the manuscript for more clarity.

3. Please correct "Province one" as "Koshi Province" in table and other places. as this province is recently named.
Authors: Thank you so much for your suggestion. We have changed "Province One" to "Koshi Province" throughout the manuscript.

4. Is this policy implications based on the findings of your study? "It is important to note that these interventions need to be culturally appropriate, and involve community participation to ensure their sustainability." If not, provide reference and try to link with your study's findings.

Authors: Thank you so much for your valuable suggestion. We have deleted the lines as per your suggestion.

5. Conceptual framework title: I think it should be like this: Conceptual framework for diarrheal risk among under two children

Authors: Thank you so much for the suggestion. We have changed the conceptual framework’s title accordingly.
